# Supplementary material for: Invertebrate composting quality of the invasive alga Rugulopteryx okamurae, prospects for its bio-recycling, management and circular economy
Source: PLoS One. 2024 Oct 23;19(10):e0311483. doi: 10.1371/journal.pone.0311483 (PMC11498720; doi:10.1371/journal.pone.0311483)
Supplement: Attachment — Submitted filename: PONE-D-23-38768_comments_2024.docx [file pone.0311483.s002.docx]

To ,

The Editor,

Plos One

I have tried my best to provide my review of the following manuscript entitled “Invertebrate composting quality of the invasive alga *Rugulopteryx okamurae*, prospects for its bio-recycling, management, and circular economy“. Although the concept seems to have some practical applications it failed to provide robust information and the manuscript is not properly structured. Furthermore, the study seems to be generalized and the paper seems to share similar conclusive results as been published by the author in other journals like: “Patón, D., García-Gómez, J.C., Loring, J. et al. Composting the Invasive Toxic Seaweed Rugulopteryx okamurae Using Five Invertebrate Species, and a Mini-review on Composting Macroalgae. Waste Biomass Valor 14, 167–184 (2023)”, thus there is nothing new in this study and it does not match the standard of PlosOne. So, in my view, the manuscript is not suitable to be accepted or published in such a high-impact factor journal. Please find the comments attached:

1. Introduction: Please consider making it precise and in flow. Many sentences lack a link between the above and the next sentences. Its difficult to make the story out of the introduction.
2. Materials and methods should be written in sections like sample collection, chemical analysis etc.. It’s difficult for a reader to extract information from paragraph-type methodology.
3. The results section is again not structured and there is no link between the flow of results. It’s very difficult to make anything out of it. Many sentences contain comments and discussion by the author which is to be restricted only in the discussion section. Also, the comparison of quality between types of composites should be based on the impact on the growth of model plant species.
4. Tables and figures should be included at the last of the manuscript.
5. Figures are not good and the graph is also blurry with no error bars. Moreover, in Figures 1 and 2 the sub-figures need to be numbered and also described in the caption separately.
