# Supplementary material for: Invertebrate composting quality of the invasive alga Rugulopteryx okamurae, prospects for its bio-recycling, management and circular economy
Source: PLoS One. 2024 Oct 23;19(10):e0311483. doi: 10.1371/journal.pone.0311483 (PMC11498720; doi:10.1371/journal.pone.0311483)
Supplement: Attachment — Submitted filename: Response_to_Reviewers.docx [file pone.0311483.s003.docx]

Response to reviewers (in green) of manuscript PONE-D-23-38768 entitled:

Invertebrate composting quality of the invasive alga Rugulopteryx okamurae, prospects for its bio-recycling, management and circular economy

Dear Dr. Patón,

Thank you for submitting your manuscript to PLOS ONE. After careful consideration, we feel that it has merit but does not fully meet PLOS ONE’s publication criteria as it currently stands. Therefore, we invite you to submit a revised version of the manuscript that addresses the points raised during the review process.

Dear Editor,

Thank you so much by this opportunity.

Please submit your revised manuscript by Feb 26 2024 11:59PM. If you will need more time than this to complete your revisions, please reply to this message or contact the journal office at plosone@plos.org. When you're ready to submit your revision, log on to https://www.editorialmanager.com/pone/ and select the 'Submissions Needing Revision' folder to locate your manuscript file.

OK

Please include the following items when submitting your revised manuscript:

A rebuttal letter that responds to each point raised by the academic editor and reviewer(s). You should upload this letter as a separate file labeled 'Response to Reviewers'.

OK

A marked-up copy of your manuscript that highlights changes made to the original version. You should upload this as a separate file labeled 'Revised Manuscript with Track Changes'.

OK

An unmarked version of your revised paper without tracked changes. You should upload this as a separate file labeled 'Manuscript'.

OK

If you would like to make changes to your financial disclosure, please include your updated statement in your cover letter. Guidelines for resubmitting your figure files are available below the reviewer comments at the end of this letter.

There were indeed some discrepancies that we have resolved in the acknowledgements. There is no need to change anything else. Thank you

If applicable, we recommend that you deposit your laboratory protocols in protocols.io to enhance the reproducibility of your results. Protocols.io assigns your protocol its own identifier (DOI) so that it can be cited independently in the future. For instructions see: https://journals.plos.org/plosone/s/submission-guidelines#loc-laboratory-protocols. Additionally, PLOS ONE offers an option for publishing peer-reviewed Lab Protocol articles, which describe protocols hosted on protocols.io. Read more information on sharing protocols at <https://plos.org/protocols?utm_medium=editorial-email&utm_source=authorletters&utm_campaign=protocols>.

The laboratory protocols that affect the maintenance of the animals are very simple to understand and we do not believe it is necessary to include them in a separate file. In addition, the laboratory analyses follow the official AOAC guidelines so the protocols are well known. Nevertheless, thank you.

We look forward to receiving your revised manuscript.

Kind regards,

Noé Aguilar-Rivera

Academic Editor

PLOS ONE

Thanks

Journal Requirements:

1. When submitting your revision, we need you to address these additional requirements.

Please ensure that your manuscript meets PLOS ONE's style requirements, including those for file naming. The PLOS ONE style templates can be found at

https://journals.plos.org/plosone/s/file?id=wjVg/PLOSOne_formatting_sample_main_body.pdf and

https://journals.plos.org/plosone/s/file?id=ba62/PLOSOne_formatting_sample_title_authors_affiliations.pdf

OK

2. Did you know that depositing data in a repository is associated with up to a 25% citation advantage (https://doi.org/10.1371/journal.pone.0230416)? If you’ve not already done so, consider depositing your raw data in a repository to ensure your work is read, appreciated and cited by the largest possible audience. You’ll also earn an Accessible Data icon on your published paper if you deposit your data in any participating repository ([https://plos.org/open-science/open-data/#accessible-data](https://plos.org/open-science/open-data/#accessible-data)).

OK. We have mentioned in the text that the data will be sent upon reasoned request. If the manuscript is finally accepted there is no objection to upload a summary of the data in a separate file. However, the laboratory that performed the analyses belongs to the CSIC (Spanish National Council of Research) and has confidentiality agreements with the University.

3. In your Methods section, please provide additional information regarding the permits you obtained for the work. Please ensure you have included the full name of the authority that approved the field site access and, if no permits were required, a brief statement explaining why.

All sample collection is done in public places and no permits are required although we inform local and regional authorities. This is mentioned in the text.

4. Thank you for stating in your Funding Statement:

All the financial support has been received by professor Jose Carlos García-Gómez and any funder have influence in the research. The details are:

- JCGG (68/83 / 4081/0171) Organization of American States (https://www.oas.org/en/).

- JCGG (68/83 / 4358/0171) Autoridad Portuaria de Sevilla (https://www.puertodesevilla.com/).

- JCGG (68/83 / 3850/0171) Diputación Provincial de Cádiz (https://www.dipucadiz.es/).

- JCGG (PRJ201903535) Fundación CEPSA (https://www.cepsa.com/es/).

- JCGG (68/83 / 3608/0171) Fundación ENDESA (https://www.endesa.com/es).

- JCGG (PRJ201903717) Red Eléctrica de España (https://www.ree.es/es).

Please provide an amended statement that declares all the funding or sources of support (whether external or internal to your organization) received during this study, as detailed online in our guide for authors at http://journals.plos.org/plosone/s/submit-now. Please also include the statement “There was no additional external funding received for this study.” in your updated Funding Statement. Please include your amended Funding Statement within your cover letter. We will change the online submission form on your behalf.

There was indeed a discrepancy between the documents. We have added the missing institutions in the acknowledgements.

5. Thank you for stating the following in the Acknowledgments Section of your manuscript:

This study was financed by the Red Eléctrica de España (REE) and CEPSA Foundation. Additional financial collaboration was provided by Diputación de Cádiz and Puerto Deportivo La Alcaidesa (La Línea). This project also benefited from scientific equipment and infrastructures financed by the Port Authority (AP) of Seville and the Aquarium of Seville

We note that you have provided funding information that is not currently declared in your Funding Statement. However, funding information should not appear in the Acknowledgments section or other areas of your manuscript. We will only publish funding information present in the Funding Statement section of the online submission form.

Please remove any funding-related text from the manuscript and let us know how you would like to update your Funding Statement. Currently, your Funding Statement reads as follows:

All the financial support has been received by professor Jose Carlos García-Gómez and any funder have influence in the research. The details are:

- JCGG (68/83 / 4081/0171) Organization of American States (https://www.oas.org/en/).

- JCGG (68/83 / 4358/0171) Autoridad Portuaria de Sevilla (https://www.puertodesevilla.com/).

- JCGG (68/83 / 3850/0171) Diputación Provincial de Cádiz (https://www.dipucadiz.es/).

- JCGG (PRJ201903535) Fundación CEPSA (https://www.cepsa.com/es/).

- JCGG (68/83 / 3608/0171) Fundación ENDESA (https://www.endesa.com/es).

- JCGG (PRJ201903717) Red Eléctrica de España (https://www.ree.es/es).

Please include your amended statements within your cover letter; we will change the online submission form on your behalf.

As mentioned above, the missing institutions have been added to the acknowledgments.

6. We note that Figure 1 in your submission contain copyrighted images. All PLOS content is published under the Creative Commons Attribution License (CC BY 4.0), which means that the manuscript, images, and Supporting Information files will be freely available online, and any third party is permitted to access, download, copy, distribute, and use these materials in any way, even commercially, with proper attribution. For more information, see our copyright guidelines: http://journals.plos.org/plosone/s/licenses-and-copyright.

We require you to either present written permission from the copyright holder to publish these figures specifically under the CC BY 4.0 license, or remove the figures from your submission:

a. You may seek permission from the original copyright holder of Figure 1 to publish the content specifically under the CC BY 4.0 license.

We recommend that you contact the original copyright holder with the Content Permission Form (http://journals.plos.org/plosone/s/file?id=7c09/content-permission-form.pdf) and the following text:

“I request permission for the open-access journal PLOS ONE to publish XXX under the Creative Commons Attribution License (CCAL) CC BY 4.0 (http://creativecommons.org/licenses/by/4.0/). Please be aware that this license allows unrestricted use and distribution, even commercially, by third parties. Please reply and provide explicit written permission to publish XXX under a CC BY license and complete the attached form.”

Please upload the completed Content Permission Form or other proof of granted permissions as an "Other" file with your submission.

In the figure caption of the copyrighted figure, please include the following text: “Reprinted from [ref] under a CC BY license, with permission from [name of publisher], original copyright [original copyright year].”

b. If you are unable to obtain permission from the original copyright holder to publish these figures under the CC BY 4.0 license or if the copyright holder’s requirements are incompatible with the CC BY 4.0 license, please either i) remove the figure or ii) supply a replacement figure that complies with the CC BY 4.0 license. Please check copyright information on all replacement figures and update the figure caption with source information. If applicable, please specify in the figure caption text when a figure is similar but not identical to the original image and is therefore for illustrative purposes only.

The aerial images are copyright free, follow open data guidelines and come from the viewer of the National Geographic Institute of Spain (https://www.ign.es/iberpix/visor/).

7. Please include captions for your Supporting Information files at the end of your manuscript, and update any in-text citations to match accordingly. Please see our Supporting Information guidelines for more information: http://journals.plos.org/plosone/s/supporting-information.

----------------------------------------

Reviewers' comments:

Reviewer's Responses to Questions

Comments to the Author

1. Is the manuscript technically sound, and do the data support the conclusions?

The manuscript must describe a technically sound piece of scientific research with data that supports the conclusions. Experiments must have been conducted rigorously, with appropriate controls, replication, and sample sizes. The conclusions must be drawn appropriately based on the data presented.

Reviewer #1: Yes

Reviewer #2: No

Reviewer #3: Partly

2. Has the statistical analysis been performed appropriately and rigorously?

Reviewer #1: Yes

Reviewer #2: No

Reviewer #3: Yes

3. Have the authors made all data underlying the findings in their manuscript fully available?

The PLOS Data policy requires authors to make all data underlying the findings described in their manuscript fully available without restriction, with rare exception (please refer to the Data Availability Statement in the manuscript PDF file). The data should be provided as part of the manuscript or its supporting information, or deposited to a public repository. For example, in addition to summary statistics, the data points behind means, medians and variance measures should be available. If there are restrictions on publicly sharing data—e.g. participant privacy or use of data from a third party—those must be specified.

Reviewer #1: Yes

Reviewer #2: No

Reviewer #3: Yes

The only restriction is the confidentiality agreement of the CSIC (Spanish National Research Council) laboratory to use the chemical data. This was added in the manuscript.

4. Is the manuscript presented in an intelligible fashion and written in standard English?

PLOS ONE does not copyedit accepted manuscripts, so the language in submitted articles must be clear, correct, and unambiguous. Any typographical or grammatical errors should be corrected at revision, so please note any specific errors here.

Reviewer #1: Yes

Reviewer #2: No

Reviewer #3: No

5. Review Comments to the Author

Please use the space provided to explain your answers to the questions above. You may also include additional comments for the author, including concerns about dual publication, research ethics, or publication ethics. (Please upload your review as an attachment if it exceeds 20,000 characters)

Reviewer #1: The paper is well-written and adds to the existing literature on the subject. The introduction is well-composed and has been developed on the right lines. The most appropriate methodology has been used for analysis of data and information. The results have been reported well. A logical sequence of interpretations has been followed and developed scientifically. The discussion has been well brought out and the cogency of arguments are well thought out. The discussion is comprehensive and complete. References are as required. May be accepted for publication

Reviewer #2: The authors present a potentially interesting bioremediation study that seeks to solve the upwelling problem of the invasive macroalgae Rugulopteryx okamurae by using its biomass for composting. The authors show the quality and composition of the composts generated by 4 different invertebrates fed with and without the invasive macroalgae Rugulopteryx okamurae algae. The study is interesting and relevant but has many issues.My first concern is the choice of statistical tests that don't seem correct. As I understand the experiment performed has 2 different factors: 1- invertebrate species (with 4 levels), and 2 - type of food, or treatment (with 2 levels), however, the authors didn't seem to consider this, as they used a simple Mann-Whitney test to compare the 2 different treatments within the same invertebrate species. Isolating the factors from the same experiment is controversial and not at all fully accepted in the scientific community. This is because it can lead to the wrong interpretation of the data. The ideal statistical test for this data would be a PERMANOVA (if non-parametric) or 2-way ANOVA (if parametric). Similarly, the use of Dunn test after the Kruskal-Wallis (I assume it was Kruskal-Wallis, because the authors only mentioned the Dunn post-hoc test) on Fig. 3 is wrong - it should, again, be either PERMANOVA or 2-way ANOVA. The other big problem I noticed here was the lack of control for this experiment. This should be a control for both food sources without the presence of any invertebrates to account for microbial decomposition.On a minor comment, the manuscript has many grammar mistakes and misspellings.With that, I believe that the manuscript as it is, is not acceptable for publication. I do think it has potential and would suggest the authors redo the experiment including the controls, and use the proper statistical tests.

We appreciate the reviewer's suggestions. However, our objective in this work was not to explore the interaction between variables by multivariate analysis, but only to determine to what extent each chemical parameter changes significantly between the two types of diet. We do not intend to explore the interaction between species and diet because these are independently managed species. The batches are experimentally separated. We only intend to find out which of the four species gives better results in terms of guano quality and if this is affected by the diet with or without algae. We did not explore interactions. However, if the evaluator considers this analysis essential, it can be done, although we believe that this would overly complicate the work for potential readers by completely departing from the proposed design.

Reviewer #3: The study sounds interesting but there is no novelty its conclusion is similar to previously published studies and the manuscript is not properly structured. Figures are not good not properly numbered and not properly described in caption. please read the .doc file

We have changed the captions of tables and figures

6. PLOS authors have the option to publish the peer review history of their article (what does this mean?). If published, this will include your full peer review and any attached files.

If you choose “no”, your identity will remain anonymous but your review may still be made public.

Do you want your identity to be public for this peer review? For information about this choice, including consent withdrawal, please see our Privacy Policy.

Reviewer #1: No

Reviewer #2: No

Reviewer #3: No

[NOTE: If reviewer comments were submitted as an attachment file, they will be attached to this email and accessible via the submission site. Please log into your account, locate the manuscript record, and check for the action link "View Attachments". If this link does not appear, there are no attachment files.]

While revising your submission, please upload your figure files to the Preflight Analysis and Conversion Engine (PACE) digital diagnostic tool, https://pacev2.apexcovantage.com/. PACE helps ensure that figures meet PLOS requirements. To use PACE, you must first register as a user. Registration is free. Then, login and navigate to the UPLOAD tab, where you will find detailed instructions on how to use the tool. If you encounter any issues or have any questions when using PACE, please email PLOS at figures@plos.org. Please note that Supporting Information files do not need this step.

Thanks

D
