# Supplementary material for: Invertebrate composting quality of the invasive alga Rugulopteryx okamurae, prospects for its bio-recycling, management and circular economy
Source: PLoS One. 2024 Oct 23;19(10):e0311483. doi: 10.1371/journal.pone.0311483 (PMC11498720; doi:10.1371/journal.pone.0311483)
Supplement: Attachment — Submitted filename: Response_to_Reviewers2.docx [file pone.0311483.s005.docx]

Dear editors and reviwers:

I respond to the corrections to the manuscript.

**Comments**

Overall, the paper effectively introduces the interesting topic of the invasive species *Rugulopteryx okamurae* and its impacts on the coast of southern Spain. It also provides a detailed and straightforward explanation of the possible alternatives to deal with this issue.

I think the article has merit and should be published. However, I included some comments per section for the authors and editor to keep in mind.

**Introduction comments**

Be aware of the use of the past tense to describe work that has been previously done. For instance, these sentences should be expressed in the past tense instead of the present tense.

In lines 31-37

That same year, 5000 mt of algae were extracted from the beaches of Ceuta (North Africa) [6]. Also in 2019, the species was detected for the first time in the Azores Islands (Portugal) [7]. In 2020, the invasion occupied more than three million m² in the Strait Natural Park [1]. In the same year, only in the municipality of Tarifa 400 mt were extracted [8]. Therefore, we can affirm that *R. okamurae* is expanding without control, which has led to its inclusion in the catalog of invasive alien species (IAS) by the Ministry for Ecological Transition

Corrected

In line 66

within the scope of circular economy

Corrected

In line 71

the reinforcement of the immune system or as neuroprotective compounds has been explored

Corrected

Consider changing this sentence in lines 83-84 as suggested for better readability

In this regard, previous research indicates that windrow systems with frequent turning can reduce waste volume by up to 83% [27].

Corrected

Consider changing this sentence in lines 86-87

Moreover [28] decrease by 39% by weight and 37% the volume of various types of waste using composting with microorganisms.

to

In other cases, a decrease of 39% by weight and 37% by volume has been achieved with various types of waste using composting with microorganisms [28].

Corrected

In line 89

which, being poikilotherms and needing to regulate their temperature,

poikilotherms are animals that can’t regulate their temperature autonomously

Corrected

In lines 110-111, the authors have this sentence:

CH4 and N2O emissions have been considered equivalent to 28 and 265 times CO2 emissions respectively

Comment: Equivalent in what sense? It’s not clear. Please, explain.

Corrected

In lines 123-124

These generate a direct impact on the environment due to their presence, but also indirectly due to the loss of sand produced by the use of excavators for cleaning, which should be replaced by rakes (personal observation).

Comment: This sentence is not well connected with the rest of the paragraph. I would consider removing it.

Corrected

**Methods**

In line 132, the authors state this

from beach outcrops (Fig 1) and stored in coolers until use for invertebrate feeding

Comment: Stored at what temperature

Corrected

In line 164

Sevilla University where the parameters Total Organic Matter (TOM, %),

Corrected

**Results**

The results are concise and clearly suggest the type of treatments that would result in a better compost quality.

OK

**Discussion**

Overall, the discussion provides a thorough overview of how using various invertebrate species for composting *R. okamurae* algae could result in potentially valuable feedstocks for agricultural purposes.

OK

**Response to previous reviewers**

I think the responses to reviewers are appropriate. However, I agree with reviewer 2 that a control without any invertebrates was not included. The authors should address this comment as it is fairly common to include uninoculated/untreated controls to account for changes not generated by the target treatment (i.e., environmental factors, microbiological factors).

In the text we have clarified that our control group is the one constituted by the feeding with 100% waste and the treatment group is the one included with 50% kitchen waste and 50% with seaweed. The seaweed alone does not compost easily due to its toxicity. In fact, months can pass and it does not finish composting as we have verified in our laboratory. Including composting with microorganisms would be a different job. We want to see the differences between invertebrates not between invertebrates and other methods.
